# In Praise of High Feeding

**Published:** 1895-12-28

**Authors:** 


### Modern Sociology.

### IN PRAISE OF HIGH FEEDING.

We have little sympathy with those exponents of the higher culture who affect to disparage a good dinner, and are horror-stricken at the introduction of food as a wholesome and instructive subject of debate. Very capable of wide application is the saying attributed to Brillat-Savarin, "Dis moi ce que tu manges, je te dirai ce que tu es." The great cook had in his mind merely individuals, but he might with equal justice have referred to communities, and even nations. Taking the human race as a whole, it may be said that it differs from the animal kingdom in that it is practically omnivorous. That man in his struggle for existence has been greatly indebted to this peculiar gift is quite beyond question. Wherever he has roamed he has found food, not always nice it is true, but still something wherewith to sustain life. The animal, however, is doomed to extinction if transported to strange climes, where its normal and hereditary tastes cannot be gratified. In our youth we were taught from elementary text-books and by easy-going pedagogues that man's food varied according to the climate—that it was a truth beyond criticism that the animal or vegetable products used as food staples in the different countries could not be improved upon as regards suitability to the inhabitants' needs. This fatal lesson—that Nature herself solves the great food problem—lingers with us to this day, albeit we recognise that if it were true, each nation would be subsisting on its own resources, and there would be no occasion for nineteen-twentieths of the world's commerce. As a matter of fact, Nature is very prone to exact from her children severe penalties from over-indulgence on her bounty. Nor is it by any means certain that a simple diet, of which the beginning, middle, and end are some valued staples, merits all the praise sometimes heaped upon it. Physiologically, such a *régime* leaves much to be desired; economically, it is the rankest of heresies.

The nation which breakfasts, dines, and sups on a single course may be mild-mannered and contented, but its material progress will be slow and its very virtues a snare. Idealists and poets have described in glowing terms the Hindoo satisfied with his rice and dahl, the Bedouin happy in the possession of the date, the South Sea Islander basking in the sun and asking for nothing better than to be left in undisputed enjoyment of his cocoanut and bread-fruit. But beneath the *couleur de rose*, in which these pictures are painted, there lurk the demons of famine and national sloth. It is quite certain in the matter of food that "too much of one good thing is good for nothing," that a superfluity of any staple is almost as dangerous to a people as a scarcity. That word-picture which we owe to Carlyle of Quashie lying under the flapdoodle-tree waiting for the flapdoodle-fruit to fall into his open mouth is no exaggerated caricature. The truth which is hidden under its sardonic grimness will be recognised by any one who has sojourned in the East or Southern Seas, or who has studied even superficially the history or economics of aboriginal types. When Nature is too bountiful, when Quashie can tickle the earth with a stick, and almost immediately look for a harvest, man's innate tendency to become omnivorous is sensibly diminished, while his disinclination to work is correspondingly increased. Nature, on the other hand, is slowly preparing for him a stern and sure corrective.

According to an old adage—

> They that have no other meat
> Bread and butter are glad to eat.

It is not a little suggestive that English folk here should regard bread and butter as synonymous with cheapness and comparative dearth. Wheaten flour is the most expensive vegetable staple known to man; its use, therefore, compared with that of rice, the banana, and the yam, implies a positive degree of national affluence. Combined with butter, it betokens that the people who regard it as the last resource of the poor have reached a very high point of gastronomic and material wealth. We confess. therefore, to only a small measure of sympathy for the fortunate citizen, keen on political economy but undisturbed with misgivings as to the state of his own larder, who periodically admonishes the British working man upon his extravagant fare, and holds up as an example to be followed the more meagre *menu* of his Continental brother. Whilst fully appreciating the culinary abilities of the French and German housewife, we feel no little satisfaction that there exists in this country less necessity than on the Continent for what may be described as domestic cheeseparing. The man who dines well habitually is able with fair grace to fall back upon something cheaper when "hard times" come; but he who has to content himself in normal days with poor and unvaried food finds, when the need for retrenchment arrives, that he is face to face with famine. Of this, India provides us with many illustrations, and so also does the distressful Sister Isle. Prior to 1846 the people of Ireland trusted almost exclusively to the potato, the most economical staple of northern climes. Consequently, when one fine morning the potato crop failed, poor Pat learned to his cost the evils of under-feeding—he had fared so badly in normal times, he could not go one worse when things were desperate. Such, we are thankful to think, will not be the experience of the British working-man, even in the very unlikely contingency of a simultaneous failure of the grain crops of the world. To the staple food of all modern nations of the front rank we in England add a plentiful supply of meat and butter and other nutritious and appetising articles. Therefore, higher prices or reduced earnings would not drive the nation so readily into famine as they would a people accustomed to more meagre fare, and that notwithstanding the fact that we depend so largely upon the outside world for "our daily bread." The question of food remains, as of yore, the most important in the world's economy; and the victory will assuredly fali to those who eat the most and spend the most upon it.

